# Broccoli, PTEN deletion and prostate cancer: where is the link?

**DOI:** 10.1186/1476-4598-9-308

**Published:** 2010-12-01

**Authors:** Giovanni Appendino, Alberto Bardelli

**Affiliations:** 1 Department Scienze Chimiche, Alimentari, Farmaceutiche e Farmacologiche, Universitá del Piemonte Orientale, I-28100, Novara; 2 Laboratory of Molecular Genetics, Institute for Cancer Research and Treatment (IRCC), University of Torino Medical School, Candiolo; 3 FIRC Institute of Molecular Oncology (IFOM), Milan; Italy

## Abstract

The concept that vegetables and fruits are relevant sources of cancer-preventive substances is strongly supported by population studies. Among others, cruciferous vegetables like broccoli, cabbage, cauliflower and Brussels sprouts are thought to affect the development of various types of cancers and especially prostate tumors. Yet, the identification of the molecular mechanisms by which the 'active' compounds contained in these vegetables mediate their anticancer activity has historically lagged behind. Accordingly, direct laboratory evidence of how individual nutrients affect cancer genes and the pathways they control remains the major obstacle to progress in this research field. Here we review a recent report investigating the interaction between sulforaphane, a dietary isothiocyanate derived from broccoli, and expression of the PTEN tumor suppressor gene in pre malignant prostate tissue.

## Introduction

The rationale for consuming fruit and vegetables to prevent cancer is that edible plants contain specific compounds or mixtures of compounds capable to reverse, suppress, prevent or delay either the initial phase of carcinogenesis or the progression of neoplastic cells to cancer [1]. This culture has been amply validated by epidemiological studies, with many intestinal cancers being currently considered as the result of a "non-deficiency malnutrition". However, little is still known on the mechanism(s) by which phytonutrients prevent cancer, and attempts to translate clinically the results of epidemiological-, cellular-, and animal studies on dietary chemopreventive compounds have so far met with limited success or with downright failure [2]. Indeed, the divide between dietary and clinical studies on cancer prevention has grown steadily. On the one hand, the awareness that fruit and vegetables are associated to a reduced incidence of cancer is well rooted, but, at the same time, there is also a wide acceptance of the skeptic and almost non-scientific view that nutrition is too complex to be summarized into molecular mechanisms of health promotion by single constituents [3]. Add also the commercial stakes from the food industry to promote the healthy properties of products enriched with ingredients that consumers can perceive as "cancer preventing", and the picture could not be more confused and confusing.

Indeed, the study of dietary phytonutrients is complicated. By definition, unlike vitamins and minerals, they are non-essential, with huge differences in individual sensibility to their biological activity. As a result, their beneficial (or detrimental) health effects are statistical in nature and can be observed only in population studies. Furthermore, the concentration of phytonutrients in food is dramatically altered by culinary processing (pealing, heating, freezing) and by a set of *nuances *not unlike those that make it possible for wine connoisseurs to distinguish a cheap wine from a precious crue (plant variety, terroir, harvesting time, processing, storing). Finally, many dietary compounds are extensively modified by phase 1 and phase 2 metabolisms, and are not present in humans in their natural form, but only as metabolites. Laboratory studies have demonstrated chemopreventive activity for several phytonutrients, and especially anthocyanins from berries, procyanidins from grape seeds, phytoestrogens from soy, isothiocyanates from cruciferous plants, epigallocatechin gallate from tea, curcumin from turmeric, and resveratrol from wine [4]. These compounds can modulate gene expression involved in the regulation of cell proliferation, differentiation, and apoptosis, as well as in the suppression of metastasis and angiogenesis. Evidence of clinical activity based on the monitoring of precise biomarkers has been reported for some of these compounds, especially with respect to colon cancer.

Sulforafane from broccoli is the best known chemopreventive isothiocyanate. It is formed from a glucosinolate precursor by enzymatic hydrolysis followed by spontaneous Lossen rearrangement. The enzyme involved (myrosinase) is inactivated by heating, but is expressed also by intestinal bacteria, so that also culinary processed broccoli maintain at least part of their chemopreventive potential.

Multiple epidemiological, mouse models and cell based studies indicate that broccoli-derived Sulforafane may affect the development of various types of cancers and especially prostate tumors.

## Discussion

So far the definition of the molecular mechanism(s) linking the consumption of Sulforafane to its antitumoral effects has been lacking. In a recently published work Traka and colleagues [5] tackled this important issue.

Their analysis is centered on how SF affects the expression profiles of tissues in relationship to the presence of the tumor suppressor PTEN gene. PTEN is a phosphatase playing an antagonist role in the phosphatidylinositol-3-kinase (PI3K/AKT) signaling pathway that controls among other biological outputs cell growth and apoptosis [6].

Why analyze the impact of SF on PTEN expression? The choice is based on a solid rationale: deletion of the PTEN gene is one of the early initiating events leading to prostatic intraepithelial neoplasia (PIN), and subsequently to cancer. Analyses from patients' samples indicate that PTEN alterations occur very frequently in human prostate cancer tissues [7]. There is also conclusive evidence that deletion of PTEN results in early onset of mPIN and subsequent development of prostate cancer in multiple mouse models. For their studies Traka and colleagues used the PTENL/L;PB-Cre4 mouse model [8]. In this mouse strain CRE-mediated inactivation of the PTEN locus allows the occurrence of PTEN deficient ('null') and wild type (WT) PTEN mice within the same litter. Once they reach the age of eight-weeks of age, pAKT is highly expressed in all epithelial prostatic cells of these mice, with most glands exhibiting mPIN.

Interestingly, the authors found that modulation of the dietary intake of SF in these mice had no effect on gene expression in mouse prostate tissue with PTEN expression, whereas in isogenic PTEN deficient tissues SF reduced PTEN deletion-mediated gene expression and induced additional changes in gene expression.

The molecular profile associated with SF treatment was highly complex and affected multiple genes involved in cell cycle arrest and apoptosis. Importantly the authors went on to show that the signature was at least partially overlapping with previously reported changes in human prostate tissue following diets enriched in broccoli (a dietary source of SF). Finally they provide preliminary evidences that the interaction between PTEN deletion and supplementation of diet with SF can result in the alternative splicing of many genes.

These tantalizing results also have drawbacks. For example, while clearly affecting the PTEN null driven transcriptional profile, SF nevertheless failed to significantly impact tumor progression as the histopathological changes induced by the knock out of PTEN were unaffected by SF. Also, it remains to be determined if the effects of SF on the expression profile of PTEN null cells are specific. For example whether SF also affects the transcriptional profile associated with inactivation of other tumor suppressor genes (such as p53) or activation of classical oncogenes (such as KRAS) remains to be shown. In other words rather then being PTEN-specific the effect of SF on transcription might be due to a general 'tumorigenic drift' of the targeted cells. Also of note, the effects of SF on exon splicing events was only measured in the mouse models, whether a diet rich in SF will have the same impact on human prostate tissues remains to be tested.

Even considering the above caveats, these results are relevant. The main reason is that they provide evidences of an alleged molecular mechanism linking the impact of a dietary component to tumor onset. As noted above, a key historical skepticism faced by the field of cancer prevention has been the lack of mechanistic evidence that natural compounds indeed affected the established cancer genes and the pathways they control. In this regard the work of Traka and colleagues represents a significant step forward as it provides a number of 'testable' hypotheses to draft molecular links between consumption of edible plants (containing specific compounds or mixtures of compounds) and the well accepted theory of cancer being 'in essence' a genetic disease.

## Abbreviations

SF: sulforaphane; mPIN: murine prostatic intraepithelial neoplasia; SF: sulforaphane.

## Competing interests

The authors declare that they have no competing interests.

## Authors' contributions

GA and AB prepared the manuscript. All authors read and approved the final manuscript.
